# Transcriptome profiling of *Gossypium barbadense* inoculated with *Verticillium dahliae* provides a resource for cotton improvement

**DOI:** 10.1186/1471-2164-14-637

**Published:** 2013-09-22

**Authors:** Yan Zhang, Xing Fen Wang, Ze Guo Ding, Qing Ma, Gui Rong Zhang, Shu Ling Zhang, Zhi Kun Li, Li Qiang Wu, Gui Yin Zhang, Zhi Ying Ma

**Affiliations:** 1 Department of Agriculture, North China Key Laboratory for Germplasm Resources of Education Ministry, Hebei Agricultural University, Baoding 071001, People’s Republic of China

## Abstract

**Background:**

Verticillium wilt, caused by the fungal pathogen *Verticillium dahliae*, is the most severe disease in cotton (*Gossypium* spp.), causing great lint losses worldwide. Disease management could be achieved in the field if genetically improved, resistant plants were used. However, the interaction between *V. dahliae* and cotton is a complicated process, and its molecular mechanism remains obscure. To understand better the defense response to this pathogen as a means for obtaining more tolerant cultivars, we monitored the transcriptome profiles of roots from resistant plants of *G*. *barbadense* cv. Pima90-53 that were challenged with *V. dahliae*.

**Results:**

In all, 46,192 high-quality expressed sequence tags (ESTs) were generated from a full-length cDNA library of *G. barbadense*. They were clustered and assembled into 23126 unigenes that comprised 2661 contigs and 20465 singletons. Those unigenes were assigned Gene Ontology terms and mapped to 289 KEGG pathways. A total of 3027 unigenes were found to be homologous to known defense-related genes in other plants. They were assigned to the functional classification of plant–pathogen interactions, including disease defenses and signal transduction. The branch of "SA→NPR1→TGA→PR-1→Disease resistance" was first discovered in the interaction of cotton–*V. dahliae*, indicating that this wilt process includes both biotrophic and necrotrophic stages. In all, 4936 genes coding for putative transcription factors (TF) were identified in our library. The most abundant TF family was the NAC group (527), followed by G2-like (440), MYB (372), BHLH (331), bZIP (271) ERF, C3H, and WRKY. We also analyzed the expression of genes involved in pathogen-associated molecular pattern (PAMP) recognition, the activation of effector-triggered immunity, TFs, and hormone biosynthesis, as well as genes that are pathogenesis-related, or have roles in signaling/regulatory functions and cell wall modification. Their differential expression patterns were compared among mock-/inoculated- and resistant/susceptible cotton. Our results suggest that the cotton defense response has significant transcriptional complexity and that large accumulations of defense-related transcripts may contribute to *V. dahliae* resistance in cotton. Therefore, these data provide a resource for cotton improvement through molecular breeding approaches.

**Conclusions:**

This study generated a substantial amount of cotton transcript sequences that are related to defense responses against *V. dahliae*. These genomics resources and knowledge of important related genes contribute to our understanding of host–pathogen interactions and the defense mechanisms utilized by *G. barbadense*, a non-model plant system. These tools can be applied in establishing a modern breeding program that uses marker-assisted selections and oligonucleotide arrays to identify candidate genes that can be linked to valuable agronomic traits in cotton, including disease resistance.

## Background

Cotton (*Gossypium* spp.) is widely cultivated because of its economically valuable fibers and oil seeds. During a plant’s life cycle, it is continuously threatened by Verticillium wilt, one of the most destructive diseases. The fungal pathogen, *Verticillium dahliae*, is extremely persistent in the soil and has a broad host range (Klosterman et al., [1]). It infects cotton roots and colonizes and occludes the xylem vessels, resulting in leaf curl, necrosis, defoliation, vascular tissue wilt, and discoloration (Additional file 1; Figure S1; Sink and Grey, [2]). A severe outbreak of this disease can reduce fiber quality and cause significant losses in yield. Between 2009 and 2010, 5.0 to 6.6 million acres, or more than 50% of the area in which cotton is grown in China, was affected (National Cotton Council of America–Disease Database).

No fungicides or other chemical means have proven effective for combating this disease. Although cultural practices such as appropriate seeding, irrigation, fertilization, and crop rotation can influence the development of this disease, none can efficiently control Verticillium wilt (Kamal, [3]). Genetic resistance is considered the most effective and sustainable management option, but has received little attention. Many crosses within certain lines of *G. hirsutum* can be readily made and multiple generations can be easily produced each year (Wang et al., [4]). However, a lack of genetics resources within such conventional breeding programs has meant that attempts to improve tolerance to Verticillium wilt have not been successful with this particular species, which accounts for >90% of the total acreage used for cotton production worldwide (Cai et al., [5]). However, plants of *G. barbadense* display high levels of wilt resistance, and could provide a good opportunity for genetically enhancing *G. hirsutum*. Despite this possibility, crosses between those species have historically resulted in abnormal separations, linkage drag, or hybrid dysgenesis. In addition, incorporating such materials from *G. barbadense* via molecular breeding techniques has been limited by a paucity of resistance genes and a lack of information about potential molecular markers. Therefore, the ability to explore functional genes or markers would be beneficial to researchers who are trying to introduce varieties with excellent wilt resistance.

Progress has been made in investigations of defense mechanisms by cotton against *V. dahliae* infection. For example, the cotton terpenoid pathway has been elucidated as an important contributor to the pathogen response by these plants (Tan et al., [6]; Luo et al., [7]; Xu et al., [8]). The phenylpropanoid pathway also has a critical protective role (Smit and Dubery, [9]; Pomar et al., [10]; Gayoso et al., [11]; Xu et al., [12]). Defense-responsive genes have been identified for the 14-3-3-like protein, PR10, anti-apoptosis (*p35*), HR-induced Hpa1Xoo, a thaumatin-like protein, major latex protein (MLP), and *GbVe* (Hill et al., [13]; Zhou et al., [14]; Wang et al., [15]; Chen and Dai, [16]; Munis et al., [17]; Tian et al., [18]; Zhang et al., [19]a). In addition, the transcriptional activator ethylene-responsive element binding factor gene (Qin et al., [20]), the oxygen transporter non-symbiotic hemoglobin gene (Qu et al., [21]), NDR1, and MAP kinase kinase (Gao et al., [22]) are associated with resistance to *Verticillium*. All of these findings indicate that various defense pathways are activated during the complicated response to *V. dahliae* by cotton. Nevertheless, although some defense-related genes have been identified from *G. hirsutum,* their molecular mechanisms remain obscure.

Several transcriptome analyses have been reported, such as from a drought-related cDNA library (Zhang et al., [23]) and an SSH library from cotton plants inoculated by *V. dahliae* (Zhang et al., [24]b). However, few studies of transcription have been described for *G. barbadense*. Although the D genome of cotton has recently been sequenced (Wang et al., [25]), its complexity means that publicly available data sets are of limited use for future research, including examinations of its entire transcriptome at specific developmental states or under certain stress conditions, as well as the elucidation of molecular mechanisms for the cotton defense response to *V. dahliae*. Such efforts would provide information on gene expression and regulation, and the amino acid content of proteins. Therefore, a thorough transcriptome analysis is essential for interpreting the functional elements of the genome and revealing the molecular constituents of cells and tissues. Extensive transcriptomic data would aid in our discovery of genes related to Verticillium wilt resistance, and would enable us to construct high density microarrays for further characterization of gene expression profiles during the cotton–*V. dahliae* interaction.

The technology used with full-length cDNA clones involves capturing mRNA via the 5’-end and stabilizing the full transcript when ligating into an appropriate vector and during reverse-transcription from the poly A tail (Carninci et al., [26]; Seki and Shinozaki, [27]). Therefore, full-length cDNA libraries can represent entire transcription units rather than partial gene sequences, making them extremely useful for transcriptome analysis and comparative genomics work (Seki et al., [28]). Such libraries also contain the transcriptional start site for most genes, and EST sequencing of the 5’-ends can reveal the untranslated region, methionine-encoding ATG codon, and translational start signal. Some researchers have used cDNA clones to construct microarrays for characterizing the binding of transcription factors (TFs) to promoter elements within the 5’-UTRs (Seki et al., [29]). Full-length cDNA clones also have a role in characterizing genetic structures in different species (Sakurai et al., [30]). Finally, as with other kinds of ESTs, results from the sequencing of such cDNA clones can be used for developing many types of genetic markers, including SSR (simple sequence repeat) and SNP (single nucleotide polymorphism) markers (Kofler et al., [31]; Galeano et al., [32]).

Because not enough genes/ESTs for *G*. *barbadense* are available in the public databases, and because the 'Pima90-53’ cultivar of this species shows significant advantages over *G. hirsutum* in both Verticillium wilt resistance and fiber quality, we decided to use full-length cDNA library construction and sequencing analysis to conduct an initial global analysis of the defense transcriptome dynamics in *G. barbadense*. Our study objectives were to create full-length cDNA libraries that would be useful for gene discovery in cotton, as well as for genome annotation and global investigations of the pathogen–plant interaction. We also proposed that the results would provide novel insights into the molecular mechanisms involved in defense processes by cotton. In doing so, we could obtain valuable resources for the development of molecular markers to study Upland cotton with recently published sequencing data for the D genome.

## Methods

### Fungal strain and inoculum preparation

The highly aggressive defoliating *Verticillium dahliae* fungal strain T5, isolated from an infected Upland cotton variety, was used for inoculation. To produce conidia, we took this strain from potato dextrose agar (PDA) plates and sub-cultured it onto Czapek’s medium (2 g of NaNO_3_, 1 g of K_2_HPO_4_, 1 g of MgSO_4_.7H_2_O, 1 g of KCl, 2 mg of FeSO_4_.7H_2_O, and 30 g L^-1^ sucrose) and incubated it at 25°C for 3 to 5 d. The resultant fungal cultures were filtered through sterile gauze to retain the mycelia. For root-dip inoculations, a conidial suspension (10^7^ mL^-1^) was prepared.

### Plant cultivation and inoculation

Seeds of cotton (*Gossypium barbadense* cv. Pima90–53) were surface-sterilized, then incubated on Petri dishes between sheets of moist filter paper for 48 h at 28°C. The new seedlings were transferred to tissue culture pots containing an MS medium (pH 6.0) supplemented with 10 g L^-1^ sucrose and 2 g L^-1^ Phytagel (Sangon Biotech Co., Ltd., Shanghai, China). They were then incubated for another 5 d at 28/25°C (day/night). Afterward, the seedlings were removed from the pots and inoculated via root-dipping for 30 s into either sterile water (for mock inoculation) or a conidial suspension of *V. dahliae*. They were transferred to fresh pots containing the same MS medium and incubated in a growth room at 25°C. The mock-inoculated and fungal-inoculated pots were placed in pairs, adjacent to each other, to minimize the effects of different microenvironments. Roots were harvested at 0, 1, 2, 4, 6, 8, 12, 24, 36, 48, 72, 96, and 120 hours post-inoculation (hpi). The treated tissues were quickly frozen in liquid nitrogen and stored at –80°C. As much mycelia as possible was removed from the roots.

Sections (approximately 0.5 mm) were cut from the surface-sterilized roots and stems of 10 seedlings per sampling time point. They were transferred to plates containing 25% PDA and incubated at 25°C for at least 2 d. They were then examined for the presence of mycelial growth from the vascular tissue; the percentage of sections showing such growth was recorded for each sample.

### Extraction and purification of total RNA

Frozen tissues were ground mechanically to a fine powder in liquid nitrogen. Total RNA was isolated with TRIzol® reagent (Invitrogen), according to the manufacturer’s guidelines. Remaining traces of genomic DNA were removed by DNase after a purification step with the CleanUp protocol of the RNeasy Plant Mini kit (Qiagen). Pellets of total RNA were re-suspended in RNAse-free water and quantified spectrophotometrically. Quality was determined via denaturing agarose gels (1.5%) that contained formaldehyde and were stained with ethidium bromide. Equal amounts of total RNA from each sampling event were pooled. The mRNA was further isolated with the PolyATract mRNA Isolation System (Promega, Madison, WI, USA).

### Library construction and EST sequencing

The cDNA was synthesized from 100 ng of mRNA with the Clontech Creator SMART cDNA synthesis system. It was recovered in autonomously replicating pSMART2IFD, using an *in vivo* fusion protocol provided by the manufacturer. Plasmid DNA was excised in *Escherichia coli* strain DH10B. Clones were picked randomly and transferred into 384-well plates. Single-pass sequencing from the 5’-end was carried out using a universal primer and BigDye Terminator, on an ABI 3730 automatic DNA sequencer (Sangon).

### Pre-processing and assembly of ESTs

All sequences were processed to remove low-quality regions and adaptor sequences, using the LUCY programs (Chou and Holmes, [33]). This step was followed by SeqClean to shorten the Poly-A/T (http://compbio.dfci.harvard.edu/tgi/software). To remove possible contamination, we screened the resulting sequences against the National Center for Biotechnology Information (NCBI) UniVec database, *E. coli* genome sequences, and *Gossypium* on ribosomal RNA sequences. EST sequences longer than 100 bp after trimming were clustered and assembled into contigs and singletons (unisequences) via EST Clustering, which was designed based on the MegaBlast and CAP3 programs (Huang and Madan, [34]; Zhang et al., [35]).

### Gene annotation

After clustering and assembly, the NCBI BLAST program version 2.2.6 (Altschul et al., [36]) was used to identify similarities between the ESTs and sequences deposited in public databases. We compared them against the GenBank non-redundant protein (Nr) and UniProt (TrEMBL and SwissProt) databases, using a cutoff E-value of 1e-5. The unigene sequences were also translated into proteins via ESTScan (Iseli et al., [37]). Translated protein sequences were then compared with information from pfam domain databases. Our Gene Ontology (GO) annotation (Harris et al., [38]) was performed with BLAST2GO (Conesa et al., [39]; Götz et al., [40]), based on sequence similarity in the UniProt databases and domains in the pfam database. WEGO (Ye et al., [41]) was used for GO functional classification of all unigenes and to plot the distribution of those same gene functions. The unigene sequences were also aligned to the Clusters of Orthologous Groups (COG) database to predict and classify functions. Pathway assignments were made based on information from the KEGG database (Kanehisa and Goto, [42]).

### Quantitative real-time PCR

For selected genes, differential gene expression was verified by Q-PCR. The roots from triplicate samples (n=3 seedlings) were collected at each time point. Gene expression analysis was performed with three biological replicates and three technical replicates. First-strand cDNA was generated from 1 μg of total RNA isolated from the roots of both mock- and *Verticillium*-inoculated *G. barbadense* 'Pima90-53’ (resistant) and *G. hirsutum* 'Han208’ (susceptible), using the Superscript First-strand Synthesis System (Invitrogen). Primers for Q-PCR were designed according to the parameters of an optimum GC content of 50%, Tm>55 to 65°C, length 18 to 30 nucleotides, and an expected amplified fragment size of 80 to 200 bp. For qPCR, 20-μL samples were run in three technical replicates on a LightCycler® Real Time PCR System (Roche Germany), using 2 μL of first-strand cDNA and SYBR Green PCR Master Mix (Takara). Amplification conditions included the following: one cycle at 94°C for 15 s; then 40 cycles at 94°C for 10 s, 59°C for 10 s, and 72°C for 15 s. Afterward, all products were subjected to melt curve analysis. A negative control without a cDNA template was run with each analysis to evaluate the overall specificity. Both *GhUBQ14* (GenBank: DW505546) and cotton *actin* (AF059484) were used as reference genes to normalize the total amount of cDNA in each reaction (Artico et al., [43]; Zhang et al., [24]b). A mean normalization from two reference genes was used to analyze the level of expression for each gene. Data from each biological replicate were given as the mean of three technical replicates. Each bar in the column chart represented the mean of three biological replicate experiments (n=3); vertical bars indicated standard errors. Relative fold-changes were calculated per the 2^-△△Ct^ method, as described by Livak and Schmittgen [44]. All of the selected genes and primers are listed in Additional file 2: Table S1.

### Transcription factor analysis

We used BLAST results for unique cotton sequences against *Arabidopsis* proteins to identify cotton sequences homologous to *Arabidopsis* TFs. The comprehensive Plant Transcription Factor Database was searched (PlantTFDB; http://planttfdb.cbi.pku.edu.cn) (Guo et al., [45]; Zhang et al., [46]a).

### Analysis of microsatellite repeats

The EST-SSRs in unigene sequences were identified by the MISA program (http://pgrc.ipk-gatersleben.de/misa), a powerful pipeline for SSR detection. The minimum repeat number was six for dinucleotides and five for tri-, tetra-, penta-, or hexanucleotides.

## Results

### Establishment of experimental system

**Figure 1:**
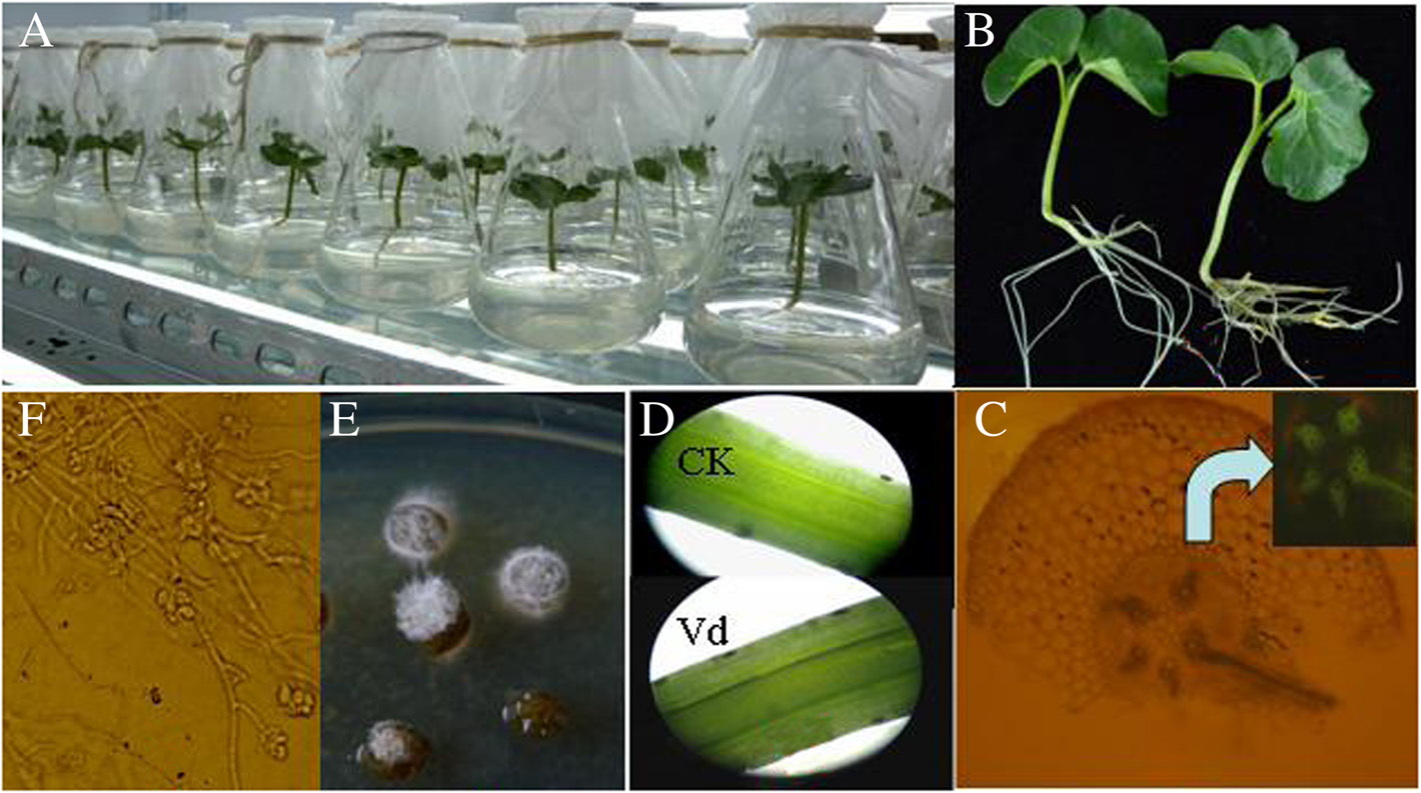
**Infection of cotton seedlings with *Verticillium dahliae*. A**: Aseptic growth in tissue culture flask. **B**: status at 3 dpi for roots treated with conidial suspension. **C**: Infection process, based on GFP-tagged *V. dahliae* strain. **D**: Vascular tissue of uninfected hypocotyl section (CK). Severe browning of vascular tissue in longitudinal section at 7 dpi. **E**: Mycelia growth in vascular tissue of surface-sterilized hypocotyl section prepared from cotton seedling at 2 dpi, then incubated for 3 d on 25% potato dextrose agar. **F**: Mycelium observed with optic microscope.

To minimize any environmental impacts, we grew the cotton seedlings in sterile culture pots and used a root dip-inoculation system for introducing the fungus. This system provided coordinated and reproducible infections under controlled conditions (Figure 1A and B). It enabled us to obtain symptoms characteristic of Verticillium wilt disease in the field. Initially, the pathogen attached to and grew on the root surface, then penetrated the roots at approximately 1 day post-infection (dpi) before entering the vascular system. It progressed through the plant to the hypocotyl vascular tissue (2 to 3 dpi; Figure 1C), where the infection was associated with he onset of vascular browning (Figure 1D). This ultimately led to wilting of the cotyledons and plant death. We monitored this movement of the pathogen by re-isolating *V. dahliae* from surface-sterilized sections of infected plants and observing the development of a GFP-tagged *V. dahliae* strain (Figure 1E and F).

To obtain a global overview of the cotton transcriptome and gene activity at nucleotide resolution, we thoroughly mixed RNA that was extracted at 1, 2, 4, 6, 8, 12, 24, 36, 48, 72, 96, or 120 hpi. To minimize any systematic bias from transcriptome sampling, and to improve our accuracy in detecting low-abundance transcripts, we constructed and sequenced three cDNA libraries from the same pooled RNA sample.

### Library construction

**Figure 2:**
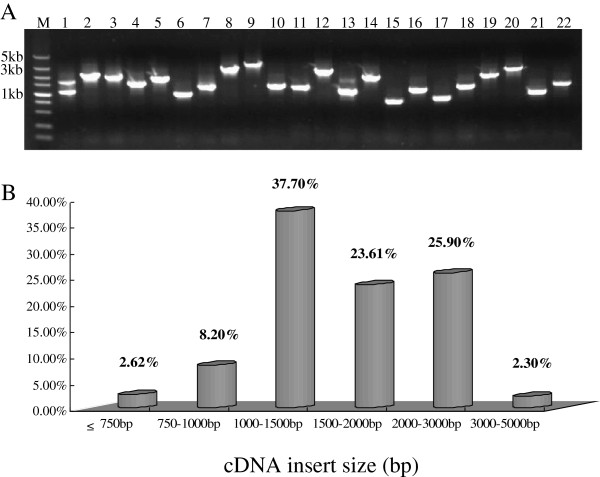
**Detection and analysis of inserts for cDNA clones.** PCR amplification of inserts for cDNA clones **(A)** and statistical analysis of insert fragment sizes in cDNA library **(B)**. Lanes: M, DL5000 marker; 1-22, random clones from library.

A high-quality full-length cDNA library was obtained. To analyze their average insert size and distribution, we randomly sampled cDNA clones from white plates. In all, 1000 clones were successfully amplified by PCR (Figure 2A). Inserts were 500 bp to 5 kb long (average 1.8 kb). As shown in Figure 1, 37.70%, 23.61%, and 25.90% of the clones had insert lengths of 1000 bp to 1.5 kb, 1.5 kb to 2.0 kb, and 2.0 kb to 3.0 kb, respectively. In addition, approximate 2.30% of the clones had inserts longer than 3.0 kb (Figure 2B). These results indicated that our full-length cDNA library contained a sufficient number of genes so that we could gain their complete sequence information.

### EST sequencing and assembly

**Figure 3:**
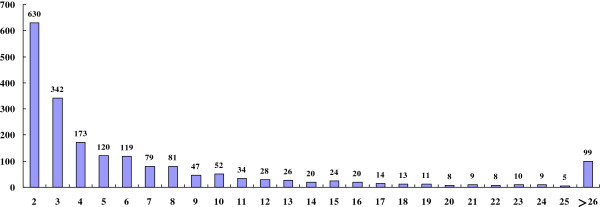
Distribution of 1981 contigs based on number of clustered ESTs.

A total of 46,192 high-quality sequences (average length 818 bp) was generated after any short and low-quality sequences were removed. Among them, 23,126 unigenes were derived from cluster assembly and sequence alignments. They included 2661 contigs and 20,465 singletons. Each contig had 2 to 1537 ESTs, with an average length of 783 bp. The majority of contigs (78.0%) contained five or fewer ESTs, while only 5.0% had 26 or more. This demonstrated low redundancy for the library. The distribution of EST numbers in each unigene indicated that several highly abundant genes could be identified, with 99 unigenes being represented by over 25 ESTs (Figure 3).

### Functional annotation of the cotton unigene dataset

A sequence similarity search was conducted against the NCBI, Nr, and SwissProt databases, using BLASTx and tBLASTn algorithms that specified E-values of less than 10^-5^. This analysis revealed that 22,446 unigenes had significant hits in the Nr database. However, of these, only 10,060 unigenes showed similarities to proteins of known function, 12,386 had similarities to predicted proteins of unknown function, and 680 unigenes had no significant similarity to any sequences contained in that database. This indicated that most of the unigenes could be assigned a known or putative function. Those without database hits were likely to include non-coding RNAs, genes whose sequences did not capture regions that contained conserved functional domains, or protein-coding genes that were novel in the database and/or cotton-specific.

### GO enrichment analysis

Based on our BLAST and SwissProt results, we further annotated the cotton unigenes with GO terms. In all, 20,397 (72.0%) could be assigned at least one term. Among them, 6,893 were placed in the category of biological processes, 8,383 in molecular functions, 11305 in cellular components, and 6184 in all three categories.

**Figure 4:**
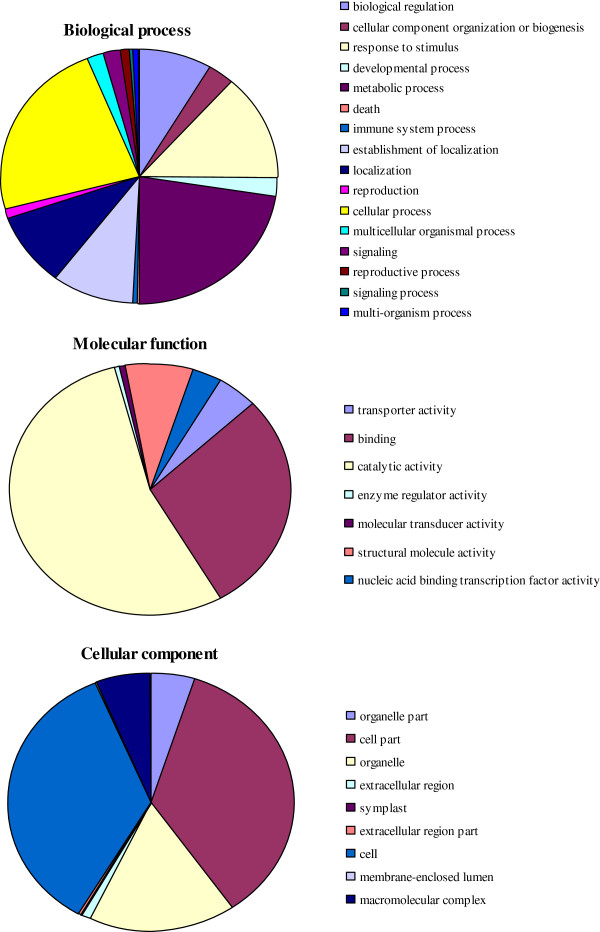
**Functional classification of unigenes from *Verticillium dahliae*-stressed cotton within categories of biological processes, molecular functions, and cellular components.**

**Figure 5:**
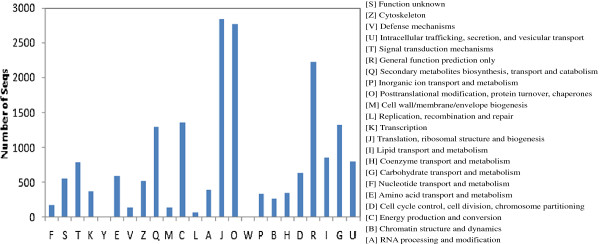
**Classifications for Clusters of Orthologous Groups (COGs).** Sequences with Nr hits were grouped into 22 COG classes.

We also used a set of plant-specific GO slims, which are a list of high-level GO terms that provide a broad overview of the ontology content. Within our three categories, the most abundant slims were for cellular processes (biological), catalytic activity (molecular), and cell part (components) (Figure 4). Metabolic and cellular processes and responses to stimulus were among the most highly represented groups within the biological-process category. The most frequent family in this library was for PR protein, followed by dirigent-like protein, and glutathione S-transferase. A large number of expressed genes were also involved in plant defense mechanisms, cytoskeleton, signal transduction, cell wall/membrane/envelope biogenesis, and lipid transport and metabolism (Figure 5).

### Pathway classification of transcripts

Using Pathway Tools (Karp et al., [47], we predicted 289 active biochemical pathways for cotton resistance to Verticillium wilt from enzyme-coding unigenes in our EST collection. Enrichment was greatest for the metabolism category (8017), followed by the categories of diseases (3190), genetic information processing (GIP; 2354), organismal systems (OS; 1901), cellular processing (CP; 1077), and environmental information processing (EIP; 773). Among the subsets for metabolism (Table 1), the major contributors were carbohydrates (24.7%), xenobiotics biodegradation and metabolism (17.1%), energy (12.4%), and amino acid metabolism (7.4%). In the GIP category, translation (60.9%), and folding, sorting, and degradation (35.0%) were the most common when compared with replication and repair (4.1%). In the EIP category, the vast majority of unigenes (98.1%) was involved in signal transduction. Transport and catabolism (42.2%), cell growth and death (36.1%), and cell communication (13.6%) constituted the majority of unigenes in the CP category. The major constituent of the organismal category was the nervous system (21.7%), endocrine system (18.4%), environmental adaptation (13.9%), and immune system (13.8%). The most important metabolite biosynthesis pathways, e.g., plant–pathogen interactions, plant hormone signal transduction, calcium signaling, and phenylpropanoid biosynthesis were well covered by our EST collection.

**Table 1:** Distribution of functions in the KEGG pathway

***Functional***		***Total***	***Percent***	***Percent***
***category***		***unigenes***	***of***	***of***
			***unigenes***	***categories***
*Metabolism (8017, 38.75%)*	Metabolism of other amino acids	729	2.74%	7/
	Xenobiotic biodegradation and metabolism	1369	5.15%	15/
	Energy metabolism	997	3.75%	8
	Carbohydrate metabolism	1981	7.45%	15/
	Terpenoid and polyketide metabolism	597	2.25%	10/
	Biosynthesis of other secondary metabolites	442	1.66%	14/
	Amino acid metabolism	737	2.77%	8
	Glycan biosynthesis and metabolism	251	0.94%	10
	Lipid metabolism	598	2.25%	12
	Nucleotide metabolism	185	0.70%	2
	Cofactor and vitamin metabolism	131	0.49%	11
*GIP (2354, 6.68%)*	Translation	1433	5.39%	5
	Folding, sorting, and degradation	825	3.10%	7
	Replication and repair	96	0.36%	7
*EIP (773, 2.91%)*	Signal transduction	758	2.85%	15
	Membrane transport	14	0.05%	2
	Signaling molecules and interactions	1	0.00%	1
*CP (1077, 4.05%)*	Cell growth and death	389	1.46%	7
	Transport and catabolism	454	1.71%	5
	Cell motility	87	0.33%	1
	Cell communication	147	0.55%	4
*OS (1901, 7.05%)*	Excretory system	126	0.47%	5
	Environmental adaptation	264	0.99%	4
	Endocrine system	349	1.31%	6
	Nervous system	413	1.55%	7
	Development	87	0.33%	3
	Immune system	263	0.99%	12
	Circulatory system	100	0.38%	2
	Digestive system	183	0.69%	8
	Sensory system	116	0.44%	3

**Figure 6:**
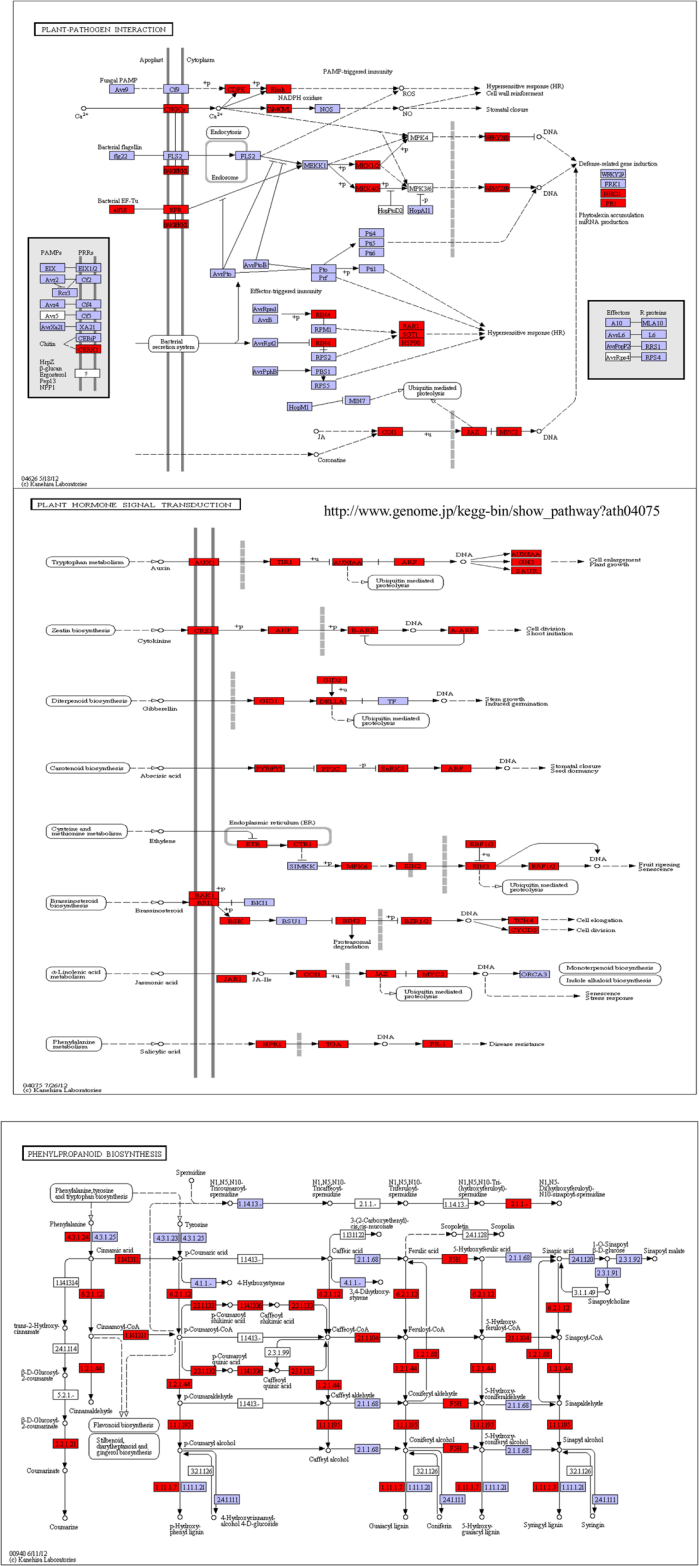
**Example of KEGG pathways found for full-length cDNA clone ESTs.** Each box shows enzymes involved in each section of pathway. Genes highlighted in red were detected from our full-length cDNA library.

Examples of KEGG pathways for plant–pathogen interactions, plant hormone signal transduction, and phenylpropanoid biosynthesis are shown in Figure 6, where genes represented in the full-length cDNA library are highlighted (i.e., Ko04626, Ko04075, and Ko00940). These three pathways served as examples of pathways influenced by pathogen attacks or various abiotic stresses.

### Comparisons of unigenes from other species of cotton and model plants

**Figure 7:**
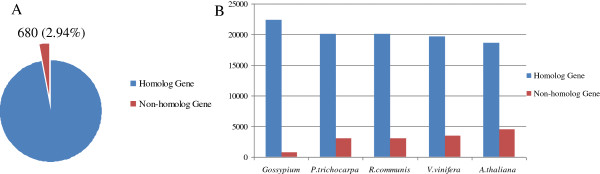
**Comparisons of 23,126 unigenes from cotton and other plant species.** Searches were performed against nucleotide databases for ESTs **(A)** or proteins **(B)** using Blastn or Blastx (E-value ≤10^-5^).

To identify which of the *G. barbadense*-specific sequences were similar to ESTs from other cotton species (*G. hirsutum*, *G. raimondii*, and *G. arboretum*), we used the unigenes as queries in a Blastn search against three databases. Overall, 680 unigenes (2.94%) had no significant matches with any sequence in the current EST databases for cotton (Figure 7A). That is, they probably are novel and belong to sequences that are specific to *G. barbadense*. In addition, we used Blastx to compare these unigenes with protein sequences from other species and found similarities of 87.1% between cotton and both *Arabidopsis* and *Populus*, 85.2% for *Vitis*, and 80.6% for *Ricinus*.

### Disease resistance-related protein families

In examining the molecular biology of a defense system in cotton, our Blast analysis determined that 3027 unigenes were homologous to known defense-related genes from other plants (Additional file 3: Table S2). They could be divided into nine major groups: perception of PAMPs by PRRs, effector-triggered immunity (ETI), ion fluxes, transcription factors, oxidative burst, pathogenesis-related (PR) proteins, programmed cell death, plant hormones, and cell wall modification (Table 2). KEGG analysis revealed that these unigenes were significantly enriched in various known resistance-relevant metabolic or signaling pathways. This indicated that such genes and pathways were highly conserved between cotton and other plants. Thus, we deduced that our unigenes were closely associated with the defense response in cotton roots against the *Verticillium* pathogen.

**Table 2:** **Known unigenes expressed in response to *V. dahliae *infection in resistant *G. barbadense***

**Pathway**	**Unigene**	**Related functions**
Perception of PAMPs by PRRs	Chitin elicitor receptor kinase (CERK1)	Chitin elicitor signaling
	Elicitor-responsive proteins (ERG)	Plant defense signaling
	Proline-rich extensin-like receptor kinases (PERKs)	Perception of PAMPs and induction of defense responses
	BRI1-associated receptor kinase 1 (BAK1)	Perception of PAMPs and induction of defense responses
	Somatic embryogenesis receptor-like kinases (SERKs)	Plant immune responses to pathogen attack
	Plant receptor-like kinases (RLKs)	Perception of PAMPs and induction of defense responses
	Mitogen-activated protein kinase (MAPK)	Downstream components in PTI
	CC-NBS-LRR resistance protein (RPM1)	Manages of signaling potential via intra-molecular negative regulation
Effector-triggered immunity (ETI)	TMV resistance protein	TMV-N mediated signal transduction pathway
	Disease resistance protein (RPS2)	Specifically recognizes effector protein from pathogen
	NBS-LRR resistance gene (RPP8)	Specifically recognizes effector protein from pathogen
	RPM1 Interacting Protein 4 (RIN4)	Negatively regulates disease resistance mediated by RPS2
Ion Fluxes	CaM-related proteins	Calcium signal transducer
	Plant cyclic nucleotide gated channels (CNGCs)	Facilitates Ca^2+^ uptake into the cytosol in response to PAMP
	Calmodulin (CaM)	Calcium signal transducer
	Calcium binding protein	Calcium signal transducer
	Calmodulin-like protein (CML)	Calcium signal transducer
	Calcineurin B-like proteins (CBL)	Decoding of calcium transients
Transcription factors (TFs)	ERF, EREBP-like	Binding ethylene-responsive element
	WRKY	Regulates signaling and transcriptional reprogramming associated with plant defense responses
	BHLH	Regulates signaling and transcriptional reprogramming associated with plant defense responses
	Histone promoter-binding protein (HBP)-1a	Negatively regulates defense response
	NADPH oxidase or respiratory burst oxidase	Generation of superoxide
Oxidative burst	Ascorbate peroxidase	Detoxifies peroxides
	Thioredoxin peroxidases	Reduces various peroxides
	Glutathione peroxidases (GPXs)	Reduces H_2_O_2_, organic hydroperoxidases, and lipid peroxides
	Cationic peroxidases	Causes a disease resistance response
	Protein disulfide-isomerase (PDI)	Ubiquitous redox protein
	Catalase	Decomposition of hydrogen peroxide to water and oxygen
	PR1C	Confers resistance to pathogen and hallmarks of defense pathways
Pathogenesis-related (PR) proteins	Beta-1,3-glucanase-like genes (PR2 homologs)	Lyses cell walls of fungal pathogens
	PR1 protein	Confers resistance to pathogen and hallmarks of defense pathways
	Chitinase (PR3 and 8 homologs)	Lyses cell walls of fungal pathogens
	Thaumatin-like protein (PR5)	Inhibits hyphal growth and sporulation by various fungi
	BAG-like genes: BCL-2-associated athanogenes	Suppresses apoptosis
	Dynamin-related proteins (DRP)	Key regulators of PCD
Programmed cell death (PCD)	Apoptosis Inducing Factor homolog (AIF)	Chromatin condensation and DNA degradation
	Nitric oxide synthase	Catalyzes arginine to produce nitric oxide
	Enhanced Disease Susceptibility 1(EDS1)	Catalyzes arginine to produce nitric oxide
	Non-expression of PR gene 1 (NPR1)	Regulatory component in SA signaling
Plant hormones	Pathogen-inducible salicylic acid glucosyltransferase (SGT1)	Involved in the early disease response and the accumulation of glucosyl SA during pathogenesis
	Phenylalanine ammonia lyase (PAL)	Key enzyme in SA biosynthesis
	Isochorismate synthase (ICS)	Key enzyme in SA biosynthesis
	Lipoxygenase (LOX)	Key enzyme in jasmonic acid (JA) biosynthesis
	Allene oxide synthase (AOS)	Key enzyme in jasmonic acid biosynthesis
	Jasmonate ZIM-motif (JAZ) proteins (TIFY10B)	JA signaling
	1-aminocyclopropane-1-carboxylic acid oxidase (ACO)	Key enzyme in ethylene biosynthesis
	3-deoxy-D-arabino-heptulosonate 7-phosphate synthase	Biosynthesis of derived secondary metabolites
Cell wall modification	Polyphenol oxidase	Oxidation of phenol compounds
	4-coumarate-CoA ligase	Phenylpropanoid metabolism
	Glutathione-S-transferase (GST)	Conjugates electrophilic molecules to glutathione (GSH)
	Caffeic acid 3-O-methyltransferase	Lignin biosynthesis
	Extension	Inhibits pathogen invasion
	Cellulose synthase	Callose synthesis
	Sucrose synthase	Sucrose synthesis
	UDP-glucuronic acid decarboxylase 1	Key factor in xylose formation

When we examined the levels of relative expression in this non-normalized cDNA library, our sequencing results (Table 3) showed that the most frequent family was PR protein 10 (1537 ESTs), followed by the dirigent-like protein (384 ESTs), glutathione S-transferase (GST-C-Tau class; 331 ESTs) and meloidogyne-induced cotton protein (331 ESTs). Other abundant families included those related to Bet_V1-like protein (275 ESTs), transmembrane protein (234 ESTs), short chain alcohol dehydrogenase (207 ESTs), gibberellin 3-beta-dioxygenase (185 ESTs), glutathione S-transferase omega (176 ESTs), and metallothionein-like type 1 protein (160 ESTs).

**Table 3:** Most frequent families found in the cotton library

***Description***	***Total ESTs***
***PR protein class 10***	1537
***dirigent-like protein***	384
***glutathione S-transferase (Tau class)***	331
***meloidogyne-induced cotton protein***	331
***Bet_V1-like protein***	275
***transmembrane protein***	234
***short chain alcohol dehydrogenase***	207
***gibberellin 3-beta-dioxygenase***	185
***glutathione S-transferase omega***	176
**metallothionein-like type 1 protein**	160

### Differential expression analysis of unigenes after *V. dahliae* inoculation

**Figure 8:**
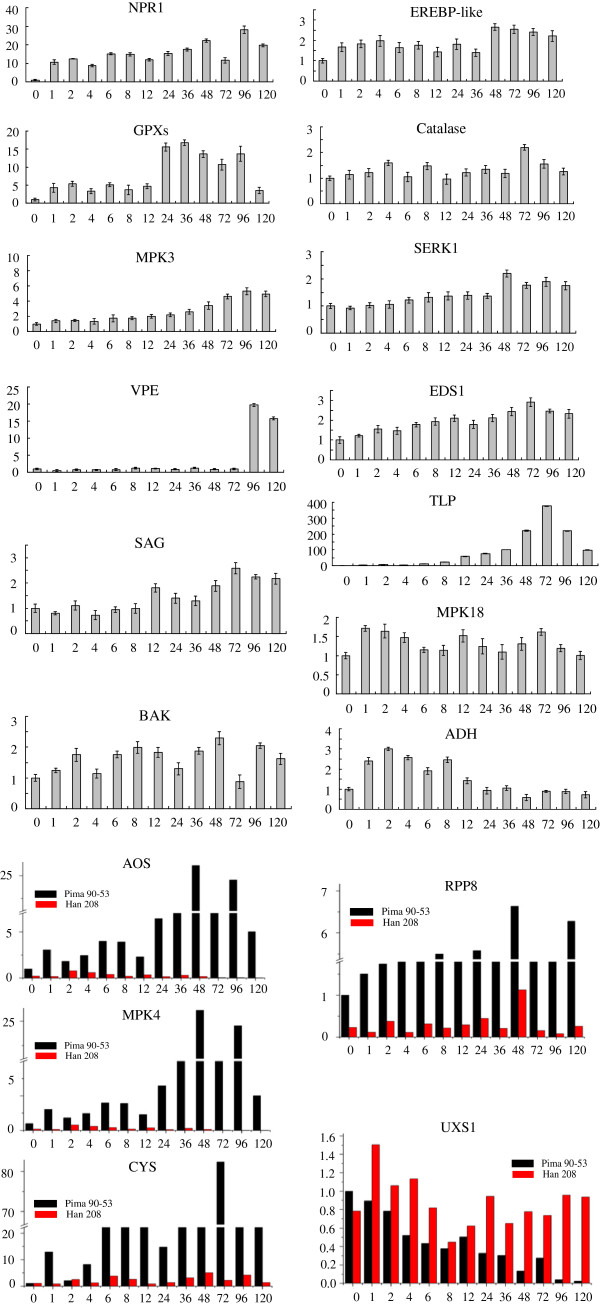
**Detailed expression profiles of defense-related genes.** Q-PCR analysis was conducted for transcription levels of selected genes in response to *V. dahliae* infection in mock-inoculated and fungal-inoculated roots at 1, 2, 4, 6, 8, 12, 24, 36, 48, 72, 96, and 120 hpi. Data within each column are means and standard errors (bar) for 3 independent Q-PCR experiments using 3 technical replicates; vertical bars indicate standard errors. Transcription level is represented as ratio of Ct value for studied gene, calibrated to mock-inoculated control and normalized to Ct value for *GhUBQ14* and cotton *actin*.

To analyze the global transcriptional changes in cotton infected with *V. dahliae*, we monitored 18 genes that function in eight metabolic pathways associated with immunity. Three patterns of expression emerged (Figure 8). First, transcripts from infected tissues could be markedly increased when compared with mock-inoculated samples. For example, the expression of TPL was rapidly induced within 2 h, with transcripts being >100-fold higher than the control at 36 hpi and peaking at >300-fold over the mock at 72 hpi. In addition, expression of *CYS*, *VPE*, *GPXs*, *NPR1*, *AOS*, and *MPK4* was 20- to 80-fold greater when compared with the mock. The second pattern was manifested by 10 genes -- *MPK3*, *MPK18*, *RPP8*, *EREBP-like*, *Catalase*, *SERK1*, *EDS1*, *ADH*, *SAG*, and *BAK* – for which transcript abundance in infected tissue was enhanced by 2- to 6-fold. The third pattern was illustrated by *UDP-glucuronic acid decarboxylase1* (*UXS1*), for which the transcription level was greatly decreased post-inoculation in the resistant *G*. *barbadense* 'Pima90-53’. Its expression was lowest at 120 hpi, being down-regulated by >10-fold. In the susceptible *G*. *hirsutum* 'Han208’, *UXS1* expression was only slightly altered, with transcripts being maintained at nearly their original level throughout the monitoring period. These differences in expression between resistant and susceptible cultivars may have reflected the degree to which they are influenced by *V. dahliae* attacks.

We subjected a subset of genes to qPCR analysis during the interactions between *V. dahliae* and the two species. The transcriptomes were compared for differentially expressed *AOS*, *MPK4*, *CYS*, and *RPP8*. After inoculation occurred, expressions were either reduced or showed delays in their upregulation in tissues from the susceptible 'Han208’ when compared with the more resistant 'Pima90-53’ (Figure 8).

### Identification of putative transcription factors

Putative TFs act as master switches by slightly modifying the expression of a series of genes. Therefore, they play critical roles in the regulation of cellular pathways during normal plant development or in response to biotic and abiotic stimuli. We identified these TFs by searching PlantTFDB2.0, a comprehensive database for 49 species that include 53,319 putative TFs and 58 families. A total of 4936 ESTs was identified in our library. The most abundant TF family was the NAC group (527), followed by G2-like (440), MYB (372), BHLH (331), bZIP (271) ERF, C3H, WRKY, C2H2, Dof, ARF, B3, GRAS, and Trihelix families (Table 4). Their distributions in *G. barbadense* and several related species are presented in Additional file 4: Table S3. Compared with other model species, frequencies were relatively higher in cotton for NAC (527; 10.67%), G2-like (440; 8.91%), C3H (265; 5.36%), Dof (169; 3.42%), s1fa-like (28; 0.57%), and STAT (13; 0.26%) but lower for ERF (270; 5.46%), HD-Zip (51; 1.03%), Wox (5; 0.1%), and ZF-HD (4; 0.08%). Except for NZZ/SPL and SAP, all of the other TF families were detected in our datasets. Yuan et al. [48] have previously reported some novel TF families within *G. barbadense*, including those for HMG, GARP-ARR-B, JUMONJI, Gif, E2F-DP, ABI3-VP1, ULT, C2C2-Dof, AS2, and ARID.

**Table 4:** **The most abundant putative transcriptional factors (TFs) in cotton resistant to *Verticillium *infection**

***TF family***	***Description***	***Total ESTs***
*NAC*	No apical meristem (NAM) protein	527
*G2-like*	Golden 2-like (GLK)	440
*MYB*	Myb-like DNA-binding domain	372
*BHLH*	Basic/helix-loop-helix domain	331
*MYB-related*	N-terminal myb-domain	321
*bZIP*	Basic leucine zipper (bZIP) motif	271
*ERF*	Single AP2/ERF domain	270
*C3H*	Zinc finger, C-x8-C-x5-C-x3-H type	265
*WRKY*	WRKY DNA-binding domain	225
*C2H2*	Zinc finger, C2H2 type	218
*Dof*	DNA binding with one zinc finger	169
*ARF*	Auxin response factor	157
*B3*	ERF-B3 family	94
*GRAS*	Three initially identified members GAI, RGA, and SCR	84
*Trihelix*	Trihelix DNA-binding domain	76
*FAR1*	Far-red-impaired Response 1	56
*HD-ZIP*	HD domain with leucine zipper motif	51
*M-type*	MADS-box transcription factors	50
*GATA*	Binds to DNA sequence "GATA"	49
*LBD*	Lateral organ boundary domain (LBD) gene family	48
*HB-other*	Homeobox domain	46
TCP	Non-canonical basic-Helix-Loop-Helix (bHLH) structure	42

### Identification of EST-SSRs

Molecular markers are valuable resources for constructing high-density genetics maps, facilitating crop breeding, and identifying traits of interest. Special EST-SSRs have high efficiency and incur a low cost, making them one of the best genetic markers for such systems. Here, 1212 SSRs were identified in 1169 (4.3%) unigenes. Among the repeat types, trinucleotide repeats were the most abundant SSRs (760, or 62.7% of the EST-SSRs), followed by dimeric SSRs (311; 25.7%), and tetrameric SSRs (98; 8.1%) (Additional file 5: Table S4). We previously reported the construction of a genome-wide map for cotton, in which these SSRs were used to add to the map density (Yang et al., [49]).

## Discussion

Because allotetraploid cotton has a large and complex genome, researchers had not previously elucidated any comprehensive sequence information to describe the transcriptome related to defense responses against *Verticillium dahliae*. Here, we investigated such responses through full-length cDNA library construction and EST sequencing. Although two tetraploid species, *G. hirsutum* and *G. barbadense*, exist, few examinations of the latter have been made because it is less commonly cultivated. However, because it is more resistant to this wilt pathogen (Zhang et al., [50]d), we used *G. barbadense* 'Pima90-53’ in our tests. As an American type of Pima, this cultivar has proven to be a better germplasm resource than other available types (i.e., Egyptian or Mid-Asian types of *G*. *barbadense*) because of its desirable traits for immunity to *Verticillium*, fiber quality, and greater yield (Ma et al., [51], Wang et al., [52]). Its resistance and phenotypic characters have long been recognized in both field and greenhouse settings. Moreover, we have previously cloned several functional genes related to resistance and/or fiber quality from 'Pima90-53’, e.g., *GbVe*, *GbWRKY1*, and *ADF1* (Chi et al., [53,54]; Liu et al., [55]; Pan et al., [56]; Zhang et al., [57]b; Zhang et al., [19]a; Zhang et al., [58]d). Therefore, we chose this specific Pima cotton for profiling the *G. barbadense* transcriptome. Our goal was to mine those genes for economically important traits so that we might potentially improve those traits in the Upland cotton *G. hirsutum*.

Infections with *V. dahliae* progress throughout the roots and into the rest of the plant, causing serious losses in both yield and quality. Because the root is the first barrier against such an attack, we selected this particular tissue for analysis. Fungal spores germinate and epidermal cells are often penetrated within the first 12 h (Fradin and Thomma, [59]). Complex perception, transduction, and exchange of signals usually occurs in the early stages of infection (Zhang and Klessig, [60]; Kunkel and Brooks, [61]; Jones and Dangl, [62]). Therefore, we sampled at 1, 2, 4, 6, 8, 12, 24, 36, 48, 72, 96, and 120 hpi to coincide with those crucial stages and to isolate early pathogen-responsive genes. We also utilized an experimental system that allowed for tight control of environmental conditions so that gene expression was not altered by any factors other than the pathogen. This enabled us to identify and monitor as many genes as possible from our library.

More than 46,192 high-quality ESTs were generated from our root cDNA library of *Verticillium*-infected *G. barbadense* seedlings. These ESTs were assembled into 23,126 unigenes. Annotation results showed that this library contained many previously reported key response genes, such as for PR protein, chitinase, members of the GST gene family, PAL, CYP, and NDR1 (McFadden et al., [63]; Li et al., [64]; Gao et al., [22]; Xu et al., [12]; Zhu et al., [65]; Ahmed et al., [66]). Our library also included several genes that function in the development of cotton fibers, e.g., *SusA1*, *CesAs*, *UXS1*, *ADF1*, *tubulin*, and *aquaporin* (Pan et al., [56]; Yuan et al., [48]; Jiang et al., [67]; Kim et al., [68]; Chi et al., [54]). Our findings suggested that defense-related genes were abundant in our library, and that genes contributing to fiber formation might also function in protecting *G. barbadense* against infection by *V. dahliae*. Therefore, this library is an important genomics resource for isolating genes with novel roles. We also demonstrated the importance of identify critical genes that code for different phenotypes, such as stress resistance or fiber quality. That is, researchers can now construct a subtracted library to find genes that are preferentially expressed in resistant lines. We previously completed an SSH library that used a resistant *G. hirsutum* cultivar and contained more than 200 genes that are differentially expressed between it and susceptible cultivars (Zhang et al. [69]). Future studies will incorporate microarrays to achieve this goal in related experiments.

When exposed to various environmental stimuli, plants utilize elaborate mechanisms to regulate cellular and molecular events so they can protect themselves with pre-formed defense barriers and induce appropriate responses. For example, pathogen-triggered immunity (PTI) constitutes the first layer of that response, restricting pathogen activity by blocking further colonization (Zhang et al., [70]). A second layer, ETI, specifically recognizes the effector by one of its NB-LRR proteins (Nürnberger et al., [71]). ETI is an accelerated response while PTI is amplified, resulting in disease resistance and, usually, a hypersensitive cell death response at the infection site. In our full-length cDNA library, groups such as LRR-RLKs, signalling-related genes, and TFs were expressed during the cotton defense response. All of them possibly contribute to these PTI- or ETI-related systems.

Most PTI-associated genes were obtained in our library, including the chitin elicitor receptor kinase (*CERK1*), which recognizes chitin oligosaccharides during plant–pathogen interactions. It acts as a representative general elicitor to induce defense responses in a wide range of plant cells (Kaku et al., [72]). We also identified chitinases, which, as PR proteins, play important roles in enhancing stress resistance in a variety of plants. These chitin-degrading enzymes hydrolyze b-(1, 4) linkages and are capable of degrading the cell walls of plant pathogenic fungi (Adams, [73]; Cheng et al., [74]; Lawrence and Novak, [75]) while releasing elicitors of defense reactions. Therefore, chitinases are thought to have crucial roles in plant defenses against biotic stresses. Based on this, we might conclude that chitinases and their elicitors confer pathogen resistance in cotton by hydrolyzing the cell walls of *V. dahliae*.

Another important PTI-related gene, *Brassinosteroid Insensitive 1-Associated Kinase 1*, or *BAK1*, was isolated here. Known as *Somatic Embryo Genesis Receptor-Like Kinase 3* (*SERK3*), it is required by PRRs (Heese et al., [76]; Dodds and Rathjen, [77]). *SERK3*/*BAK1* not only interacts with *BRI1*, which is involved in brassinosteroid signal transduction, but it also rapidly forms a complex with *FLS2*, which functions in reactions for plant disease resistance. For example, the *BAK1*/*SERK4* homolog in *Nicotiana* has a direct role in elicitor perception of bacterial cold shock protein, flagellin, and elicitin, but not chitin (Heese et al., [76]). However, we must still investigate whether similar elicitors exist in *V. dahliae* and how the *FLS2*–*BAK1* complex acts to confer pathogen immunity in resistant cotton plants.

In addition to the genes already mentioned, we obtained another important clue regarding wilt resistance in cotton. The major pollen allergen (Bet v1) family protein was one of the most abundant in our library. Proteins in this family have previously been identified under various biotic and abiotic stresses in birch, potato, pea, soybean, and cotton (Breiteneder et al., [78]; Matton and Brisson, [79]; Barratt and Clark, [80]; Crowell et al., [81]; Cheng et al., [74]). Family members may also have essential roles in the defense response by Sea Island cotton to Verticillium wilt (Chen et al., [16]). The mRNA transcripts examined in our study demonstrated that Bet v1 family genes were more abundant than any others, suggesting that this family is a vital component of the cotton response to *Verticillium* infection.

With critical roles in complex signaling cascades, phytohormones have been integrated into current models for defense responses (Bari and Jones, [82]; Grant and Jones, [83]). For example, the pathways for salicylic acid (SA), jasmonic acid (JA), ethylene (ET), and brassinosteroids are important regulators of expression by defense-related genes (Bari and Jones, [82]). In general, SA induces systemically acquired resistance and is implicated in plant tolerance to biotrophic pathogens (Spoel and Dong, [84]; Leon-Reyes et al., [85]). Both ET and JA are typically associated with defense responses to necrotrophic pathogens (Spoel et al., [86]; Bari and Jones, [82]). Cross-talk can occur between those SA- and JA/ET-mediated defense reactions to abiotic- and biotic-stress stimuli (Grant and Jones, [83]). Results from our Q-PCR analysis showed that expression patterns were the same for *EDS1* and *PAD4*. Both genes were up-regulated upon inoculation, and their transcript levels were several-fold higher than that of the mock. This implied that the *EDS1*–*PAD4* complex interferes with activation of innate cotton immunity. In addition, while identifying the biochemical pathways that were active during the response to *V. dahliae* inoculation, we discovered a plant hormone signal transduction pathway, within which exists an important branch: "SA→NPR1→TGA→PR-1→Disease resistance". The presence of this branch might suggest its role in the resistance response. However, in contrast to earlier conclusions (Leon-Reyes et al., [85]), we determined that SA is not involved in resistance to necrotrophic pathogens, such as *V. dahliae*. Therefore, we deduced that the process by which cotton becomes infected by *Verticillium* involves both biotrophic and necrotrophic stages.

The plant cell wall serves not only as a physical barrier, but as a defense barrier against pathogen penetration. Secondary metabolites play a fundamental role in the plant’s ability to fight against invading pathogens (Dubery and Smit, [87]; Naoumkina et al., [88]). That capacity is derived through multiple pathways, including those for the biosynthesis of phenylpropanoids, terpenoids, and cellulose. Most of the genes associated with those pathways, e.g., genes for caffeic acid, 3-O-methyltransferase, glutathione S–transferase, 4-coumarate:CoA ligase, UDP-glucuronic acid decarboxylase, cellulose synthase, and sucrose synthase. In this study, we detected a large phenylpropanoid pathway that encompassed those for flavonoid and lignin biosynthesis. We also obtained most of the genes that encode enzymes involved in the lignin pathway. For example, PAL, located in the core and entry of the phenylpropanoid pathway, is responsive to both biotic and abiotic stresses, including pathogen attack, and wounding (Huang et al., [89]). Likewise, POD has a role in reinforcing cell walls against the effects of pathogens or wounding through the polymerization of monolignols into lignin (Marjamaa et al., [90]). In *Arabidopsis*, Laccase4 and Laccase17 contribute to the constitutive lignification of stems, with the latter being involved in the deposition of G lignin units in fibers (Berthet et al., [91]). Therefore, all of these findings demonstrate that the phenylpropanoid pathway has an essential role in preventing the invasion or expansion of pathogens by reinforcing the cell wall. Nonetheless, future research should focus on the exact function of related genes within the phenylpropanoid pathway. Further characterization of those genes may provide valuable candidates for efforts toward the genetic improvement of cotton.

## Conclusion

In this study, we characterized the root transcriptome of *G. barbadense* and provided gene resource that related to defense responses against *V. dahliae*. These findings provide a substantial contribution to existing sequence resources for cotton, and a strong basis for future genomic research. The putative signaling pathways generated in the present study revealed that the defense system of cotton may be a complex process. The findings of this study will hopefully accelerate research on resistance in cotton to *V. dahliae* and contribute to a better understanding of the cotton defense response to plant pathogens. Besides, the spatial and temporal expressions of defense-related genes require further study.

## Competing interests

The authors declare that they have no competing interests.

## Authors’ contributions

MZY conceived the experiment, coordinated and supervised the research, and drafted the manuscript. ZY and WXF had the main responsibility for full-length library construction and writing the manuscript. DZG, MQ, and ZGR were in charge of RNA isolations and assessment of RNA quality; ZSL assisted in the cultivation of *Verticillium dahliae* and sampling the tissues. ZY, ZGR, and LZK assisted in gene selection, RT-qPCR, and statistical analyses. WLQ and ZGY assisted in our sequence analysis. All authors read and approved the final manuscript.

## Supplementary Material

Additional file 1: Figure S1 Cotton naturally grew in the field. **(A)***G. barbadense* cv. Pima90-53 displayed excellent resistance against *V. dahliae.***(B)***G. hirsutum* cv. Han208 naturally infected Verticillium wilt in the field. **(C)** Severe browning of vascular tissue in a longitudinal section of infected plants. **(D)** Typical sectorial necrosis from which *V. dahliae* mycelium may be re-isolated **(E)**. Monospore was cultured on 25% potato dextrose agar. (**F)** Mycelium of *V. dahliae* was observed by optic microscope. Click here for file

Additional file 2: Table S1 The unigenes used in Q-PCR analysis. Click here for file

Additional file 3: Table S2 Unigenes of known disease/stress response functions discovered in response to *V. dahliae* infection in resistant *G. barbadense* cv. Pima90-53. Click here for file

Additional file 4: Table S3 The most frequent sequences in our cDNA library. Click here for file

Additional file 5: Table S4 Number of dinucleotide, trinucleotide and tetranucleotide repeats. Click here for file
