# Supplementary material for: Transcriptome profiling of Gossypium barbadense inoculated with Verticillium dahliae provides a resource for cotton improvement
Source: BMC Genomics. 2013 Sep 22;14:637. doi: 10.1186/1471-2164-14-637 (PMC3849602; doi:10.1186/1471-2164-14-637)
Supplement: Additional file 1: Figure S1 — Cotton naturally grew in the field. (A)G. barbadense cv. Pima90-53 displayed excellent resistance against V. dahliae.(B)G. hirsutum cv. Han208 naturally infected Verticillium wilt in the field. (C) Severe browning of vascular tissue in a longitudinal section of infected plants. (D) Typical sectorial necrosis from which V. dahliae mycelium may be re-isolated (E). Monospore was cultured on 25% potato dextrose agar. (F) Mycelium of V. dahliae was observed by optic microscope. [file 1471-2164-14-637-S1.doc]

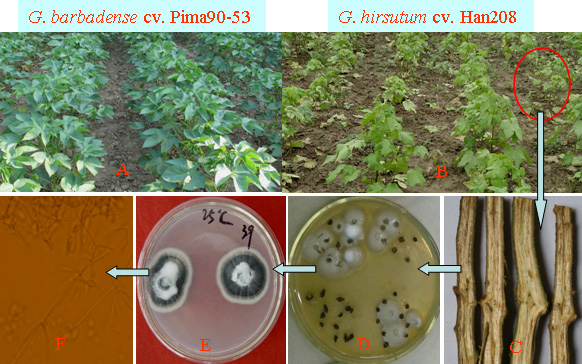


Figure S1. Cotton naturally grew in the field. (A) *G. barbadense* cv. Pima90-53 displayed excellent resistance against *V. dahliae.* (B) *G. hirsutum* cv. Han208 naturally infected Verticillium wilt in the field. (C) Severe browning of vascular tissue in a longitudinal section of infected plants. (D) Typical sectorial necrosis from which *V. dahliae* mycelium may be re-isolated (E). Monospore was cultured on 25% potato dextrose agar. (**F)** Mycelium of *V. dahliae* was observed by optic microscope.
