# Supplementary material for: Transcriptome profiling of Gossypium barbadense inoculated with Verticillium dahliae provides a resource for cotton improvement
Source: BMC Genomics. 2013 Sep 22;14:637. doi: 10.1186/1471-2164-14-637 (PMC3849602; doi:10.1186/1471-2164-14-637)
Supplement: Additional file 2: Table S1 — The unigenes used in Q-PCR analysis. [file 1471-2164-14-637-S2.doc]

Table S1 The unigenes used in Q-PCR analysis

| Unigene name | Primer sequence | Pathway |
| --- | --- | --- |
| NPR1 | F: AATGGCTGGAGATGTTTCTGTTGC  R:CGAACGCAATATGACAAACCACTTAG | Programmed cell death (PCD) |
| EREBP-like | F:TCACTGCTTCCACTGATTTTACCTC  R:GTAGAGTTATCGGACGGTGCCCA | Transcription factors (TFs) |
| GPXs | F:GGGGGACGACATCAAGTGGAAT  R: CAAAGCCTGAGGTAACGAAGCC | Oxidative burst |
| Catalase | F:CCTGTCTTCGTCTACCTCGCCT  R:TTCTGGAATCCGCTCCCTGTCA | Oxidative burst |
| MPK3 | F:GCTTATCTTGAAAGGTTACACGACA  R:CATCAGCCACCACACTCTTACATC | Perception of PAMPs by PRRs |
| SERK1 | F:GACACAAACTGCTCGTAAAGGACA  R:CGTCGCTACCGTTACATTCACCA | Perception of PAMPs by PRRs |
| VPE | F:GACGAGAACTCCTTGTTGCCACCT  R:GCTTTTGGGCTTCAGTCTTCCTAAC | Programmed cell death (PCD) |
| EDS1 | F:AATGAAGATTGATGGGTCGGAGTTAC  R:TTAGTGTGTCCCAAGGGCAACGATA | Programmed cell death (PCD) |
| SAG | F:CCCGCTTCAAGTTTCAGAGTTAC  R:CGTTCCAGAATCTTGACATCCAG | Plant hormones |
| TLP | F:GACTTCATTGACATCTCCAACATCG  R:GTCGGTAGGTTGACAGTTCCCAG | Pathogenesis-related (PR) proteins |
| BAK1 | F:GCGTGATTGGGTAAAAGGACTTCTAA  R:CCACTTCAGACATCTTCGGTCGTTC | Perception of PAMPs by PRRs |
| MPK18 | F:GGGTGTTTCCTTTATCTTGGGTTGA  R:TTGCCACTTTCTCCCCAGTATGC | Perception of PAMPs by PRRs |
| ADH | F:CACCTGGGAAAAAAGTGGGAATAG  R:CGGAGAAGTGCTGATGACGGTTAC | Cell wall modification |
| AOS | F:GCACCTTTTCACTGGCACTTACAT  R:CGGCAACACTCGGTCTCTACTTA | Plant hormones |
| MPK4 | F:CTGAGATGTGATTTGCTCCCCCA  R:GGCATTACCGATTTTCTTGATAGC | Perception of PAMPs by PRRs |
| CYS | F:TTGCTTGACTGTCCATTGTGCTACG  R:GAAAGTTCGATCCCTTGTGGCTTG | Programmed cell death (PCD) |
| UXS1 | F:CTACGATGAGGGAAAACGAGTGG  R:ATTGCGGAGTGCTTGACCTATGA | Cell wall modification |
| RPP8 | F:TCCAGAGGGATTGAGGTTCATTAC  R: TTCTCCTCCTTCCTCCAATCTATC | Effector-  triggered immunity (ETI) |
