# Supplementary material for: Transcriptome profiling of Gossypium barbadense inoculated with Verticillium dahliae provides a resource for cotton improvement
Source: BMC Genomics. 2013 Sep 22;14:637. doi: 10.1186/1471-2164-14-637 (PMC3849602; doi:10.1186/1471-2164-14-637)
Supplement: Additional file 3: Table S2 — Unigenes of known disease/stress response functions discovered in response to V. dahliae infection in resistant G. barbadense cv. Pima90-53. [file 1471-2164-14-637-S3.doc]

**Table S2** Unigenes of known disease/stress response functions discovered in response to *V. dahliae* infection in resistant *G. barbadense* cv. Pima90-53.

| Unigenes name | annotation |
| --- | --- |
| Elicitor-responsive protein | Elicitor-responsive protein 1 |
| CERK1 | Chitin elicitor receptor kinase (CERK1) |
| PBS1 | Serine/threonine-protein kinase PBS1 |
| BAK1 | BRASSINOSTEROID INSENSITIVE 1-associated receptor kinase 1 |
| SERK1 | Somatic embryogenesis receptor kinase 1 |
| MPK1 | Mitogen-activated protein kinase 1 |
| MPK3 | Mitogen-activated protein kinase 3 |
| MPK4 | Mitogen-activated protein kinase 4 |
| MPK8 | Mitogen-activated protein kinase 8 |
| MPK6 | Mitogen-activated protein kinase 6 |
| MPK16 | Mitogen-activated protein kinase 16 |
| MPK20 | Mitogen-activated protein kinase 20 |
| MPK18 | Mitogen-activated protein kinase 18 |
| Mitogen-activated protein kinase kinase .kinase A | Mitogen-activated protein kinase kinase A |
| MEKK3 | Mitogen-activated protein kinase kinase kinase 2 |
| PBS1 | Serine/threonine-protein kinase PBS1 |
| RIN4 | RPM1-interacting protein 4 |
| EDR2 | enhanced disease resistance protein (EDR2) |
| NBS | NBS resistance protein-like protein |
| NBS | NBS type disease resistance protein |
| NBS | nbs-lrr resistance protein |
| RPM1 | Disease resistance protein RPM1 |
| IRAK4 | interleukin-1 receptor-associated kinase 4 |
| Calmodulin | Calmodulin |
|  | calcium-binding protein CML |
| Calmodulin-like protein | Calmodulin-like protein 11 |
| CBL | Calcineurin B-like protein 3 |
|  | Calcineurin B-like protein 3 |
|  | Calcineurin B-like protein 8 |
|  | Calcineurin B-like protein 10 |
|  | Calcineurin B-like protein 1 |
| CIPK23 | CBL-interacting serine/threonine-protein kinase 23 |
| CIPK7 | CBL-interacting serine/threonine-protein kinase 7 |
| CIPK6 | CBL-interacting serine/threonine-protein kinase 6 |
| CIPK11 | CBL-interacting serine/threonine-protein kinase 11 |
| CIPK | CBL-interacting serine/threonine-protein kinase |
| CNGC | Cyclic nucleotide-gated ion channel |
| CNGC13 | Cyclic nucleotide-gated ion channel 13 |
| CNGC1-LIKE | cyclic nucleotide-gated ion channel 1-like |
| CNGC1 | Cyclic nucleotide-gated ion channel 1 |
| CNGC5 | Probable cyclic nucleotide-gated ion channel 5 |
| voltage-gated potassium channel | Probable voltage-gated potassium channel subunit beta |
| Chloride channel protein CLC-b | chloride channel protein CLC-c |
|  | Chloride channel protein CLC-b |
| WRKY2 | WRKY transcription factor 2 |
| WRKY6 | WRKY transcription factor 6 |
| WRKY26 | Probable WRKY transcription factor 26 |
|  | Probable WRKY transcription factor 26 |
| WRKY29-4 | Probable WRKY transcription factor 29-4 |
| WRKY6 | Probable WRKY transcription factor 6 |
| WRKY11 | Probable WRKY transcription factor 11 |
| WRKY transcription factor | putative WRKY transcription factor |
| WRKY 17 | WRKY transcription factor 17 |
| WRKY 1 | transcription factor WRKY1 |
| WRKY28 | Probable WRKY transcription factor 28 |
| WRKY30 | WRKY transcription factor 30 |
| WRKYB | WRKY transcription factor B |
| WRKY23 | WRKY transcription factor 23 |
| WRKY22 | WRKY transcription factor 22 |
| WRKY46 | WRKY transcription factor 46 |
| WRKY31 | Probable WRKY transcription factor 31 |
| WRKY33 | Probable WRKY transcription factor 33 |
| WRKY40 | Probable WRKY transcription factor 40 |
| WRKY60 | Probable WRKY transcription factor 60 |
| WRKY65 | Probable WRKY transcription factor 65 |
|  | Probable WRKY transcription factor 65 |
| WRKY72 | Probable WRKY transcription factor 72 |
| WRKY70 | Probable WRKY transcription factor 70 |
| WRKY23-1 | Probable WRKY transcription factor23-1 |
| WRKY75 | Probable WRKY transcription factor 75 |
| BHLH18 | Transcription factor bHLH18 |
| BHLH25 | Transcription factor bHLH25 |
| BHLH60 | Transcription factor bHLH60 |
| BHLH149 | transcription factor, putative |
| BHLH47 | Transcription factor bHLH47 |
| BHLH68 | Transcription factor bHLH68 |
| BHLH147 | Transcription factor bHLH35 |
|  | Transcription factor bHLH35 |
| BIM2 | Transcription factor BIM2 |
| EREBP-like factor | EREBP-like factor |
| ethylene-responsive transcription factor 1 | AP2/ERF domain-containing transcription factor |
| ERF | ethylene-responsive transcription factor 1 |
|  | Ethylene-responsive transcription factor 1A |
|  | Ethylene-responsive transcription factor 1B |
|  | Ethylene-responsive transcription factor 1B |
|  | Ethylene-responsive transcription factor 1B |
|  | Ethylene-responsive transcription factor 1B |
|  | Ethylene-responsive transcription factor 1B |
|  | Ethylene-responsive transcription factor 11 |
| transcription factor TGA | Transcription factor HBP-1b |
| plant G-box-binding factor | BZIP domain class transcription factor |
| Cationic peroxidase | Cationic peroxidase 1 |
|  | Cationic peroxidase 2 |
|  | Cationic peroxidase 2 |
| Glutathione peroxidases (GPXs) | Probable phospholipid hydroperoxide glutathione peroxidase |
|  | Probable phospholipid hydroperoxide glutathione peroxidase 6 |
| ascorbate peroxidases | L-ascorbate peroxidase 3, peroxisomal |
|  | L-ascorbate peroxidase, cytosolic |
| catalase | Catalase isozyme 1 |
|  | Catalase |
|  | Catalase isozyme 2 |
| cinnamyl-alcohol dehydrogenase (ADH) | cinnamyl alcohol dehydrogenase 3 |
| Alcohol dehydrogenase | Probable cinnamyl alcohol dehydrogenase 1 |
| Pathogenesis-related protein  (PR) | Pathogenesis-related protein PR-10 |
|  | thaumatin-like protein |
|  | pathogenesis-related protein 1 |
|  | Pathogenesis-related protein 1C |
|  | Pathogenesis-related protein PR-4A |
| beta-1,3-glucanase | Glucan endo-1,3-beta-glucosidase 14 |
|  | beta-1,3-glucanase |
| Glucan endo-1,3-beta-glucosidase (glc) | Glucan endo-1,3-beta-glucosidase 5 |
|  | Glucan endo-1,3-beta-glucosidase GII |
|  | Glucan endo-1,3-beta-glucosidase, basic vacuolar isoform |
|  | Glucan endo-1,3-beta-glucosidase7 |
| chitinase | chitinase |
| Thaumatin-Like Protein (TLP) | Thaumatin-like protein |
| Metacaspase-4 | Metacaspase-4 |
| Metacaspase-1 | Metacaspase-1 |
| Defender against apoptotic cell(dad1) death (DAD1) | Defender against cell death 1 |
| Apoptosis Inducing Factor (AIF) | apoptosis-inducing factor homolog A-like |
| Dynamin-related proteins (DRP) | Dynamin-related protein 12A |
|  | Dynamin-related protein 3A-like |
| nitric oxide synthase (NOS) | Nitric oxide synthase-interacting protein |
| Arginine decarboxylase | Arginine decarboxylase |
| Non-expressor of PR gene 1 (NPR1) | Regulatory protein NPR1 |
| Pathogen-inducible salicylic acid glucosyltransferase (SAG) | pathogen-inducible salicylic acid glucosyltransferase |
| phenylalanine ammonia lyase (PAL) | Phenylalanine ammonia-lyase |
|  | Phenylalanine ammonia-lyase 1 |
|  | linoleate 9S-lipoxygenase |
| Allene oxide synthase (AOS) | Allene oxide synthase 2 |
| AOS | hydroperoxide dehydratase |
| ethylene insensitive-like protein 3 | Protein ETHYLENE INSENSITIVE 3 |
| JAZ | Protein TIFY 10A |
| TIFY10B (JAZ) | Protein TIFY 10B |
| JAZ | Protein TIFY 3B |
| 1-aminocyclopropane-1-carboxylic acid oxidase (ACO) | 1-aminocyclopropane-1-carboxylate oxidase |
| 3-deoxy--D-arabino-heptulosonate 7-phosphate synthase (DAHPS) | Phospho-2-dehydro-3-deoxyheptonate aldolase 2, chloroplastic |
|  | Phospho-2-dehydro-3-deoxyheptonate aldolase 1, chloroplastic |
| 4-coumarate--CoA ligase (4CL) | 4-coumarate--CoA ligase |
| polyphenol oxidase (PPO) | Polyphenol oxidase, chloroplastic |
| extension | Extensin-like protein |
| Caffeic acid 3-O-methyltransferase (COMT) | Caffeic acid 3-O-methyltransferase |
| HSP90-like gene | Heat shock protein 90 |
| UDP-glucuronic acid decarboxylase 1 | UDP-glucuronic acid decarboxylase 1 |
| cellulose synthase | Probable cellulose synthase A catalytic subunit 2 [UDP-forming] |
| glutathione-S-transferase(GST) | Probable glutathione S-transferase theta, gst |
|  | Probable glutathione S-transferase tau, gst |
